# Implementing adaptive youth-centered adolescent sexual reproductive health programming: learning from the Adolescents 360 project in Tanzania, Ethiopia, and Nigeria (2016-2020)

**DOI:** 10.12688/gatesopenres.13589.1

**Published:** 2022-03-25

**Authors:** Matthew Wilson, Meghan Cutherell, Abednego Musau, Sara Malakoff, Alexis Coppola, Metsehate Ayenekulu, Edwin Mtei, Fifi Ogbondeminu

**Affiliations:** 1 Population Services International (PSI), Washington, DC, 20036, USA; 2 Society for Family Health (SFH) Nigeria, Abuja, Nigeria

**Keywords:** AYSRH, Enabling Environment, Adaptive Implementation, Contraceptive Continuation, Sustainability, A360, Aspirational Programming, HCD

## Abstract

Adolescents 360 (A360) was a 4.5-year project working directly with young people to increase demand for, and voluntary uptake of, modern contraception among adolescent girls aged 15 to 19 years. A360 utilized human centered design (HCD) to create four adolescent sexual and reproductive health (ASRH) interventions across three countries - *Smart Start *in Ethiopia, *Kuwa Mjanja* in Tanzania, *Matasa Matan Arewa (MMA) *in northern Nigeria, and *9ja Girls *in southern Nigeria. A360’s interventions tap into girls’ aspirations and position contraception as a tool that can support them in pursuing their life goals. As A360 transitioned from its first program phase into its follow-on in 2020, the project examined what it had accomplished, where it had failed, and what it had learned in the process, with the goal of contributing to the global evidence base and building on these lessons in its follow-on program. A360 draws out five key lessons in this publication. These lessons speak to 1) the value of A360’s **aspirational program components** and the need to meaningfully support girls to pursue their life goals holistically; 2) the necessity of taking a consistent and rigorous approach to improving the **enabling environment** for contraceptive use to promote transformative change; 3) the need to find program and measurement approaches that respond to girls’ unique patterns of sexual activity, and support **contraceptive continuation**; 4) the usefulness of **continuous program improvement** during implementation to maintain a user-centered focus and create a culture of curiosity and innovation; and 5) the tension between designing for users and **beginning with program sustainability in mind** from the outset. A360 continues to grow in its understanding of what it takes to support sustained, transformative, holistic change for adolescent girls and commits to openness and transparency regarding successes and failures during its next project phase.

## Disclaimer

The views expressed in this article are those of the authors. Publication in Gates Open Research does not imply endorsement by the Gates Foundation.

## Background

As of 2019, an estimated 261 million young women aged 15–19 years live in low- and middle-income countries (LMICs) and represent 16% of all women of reproductive age (15–49) ^1^. By 2030, the number of adolescent women aged 15–19 years in LMICs will increase to 286 million ^2^. Yet the specific needs of adolescents are often neglected or underemphasized in global, regional, and national-level priorities, particularly their sexual and reproductive health (SRH) needs. Adolescents aged 15 to 19 have a low contraceptive prevalence rate (CPR), estimated to be around 10.2% ^3^. There is limited data on contraceptive prevalence among younger adolescents aged 10–14 ^1, 4^. Adolescents also have nearly double the unmet need of all women of reproductive age women (43% vs. 24%) ^1^. In many countries, there are tremendous gaps between age at first sex and age at first contraceptive use. In Nigeria, for example, where the median age at first sex is 17 years, women experience a gap of nearly eight years between first sex and first contraceptive use, and by the time of first contraceptive use, women have on average three or more children ^5^.

Lack of SRH knowledge and access to comprehensive SRH services, and cultural norms that promote early marriage and childbearing all contribute to these disparities ^6^. Of the 21 million pregnancies among 15 to 19 year-olds in low- and middle-income countries in 2019, half were unintended; of these, over half (55%) ended in abortion, often unsafe ^1^. Pregnancy and childbirth complications are the leading cause of death among girls aged 15 to 19, and children born to adolescent mothers face higher risk of low birth weight, neonatal complications, and other long-term adverse effects ^6^. Unmarried adolescent mothers experience socioeconomic consequences such as stigma, isolation, and discontinued schooling, placing their future financial prospects in jeopardy, and perpetuating intergenerational poverty ^7– 9^.

### Adolescents 360

In 2016, Population Services International (PSI) and its consortium of partners ^i^ launched Adolescents 360 (A360) with funding from the Bill & Melinda Gates Foundation (BMGF) and the Children’s Investment Fund Foundation (CIFF). A360 was a 4.5-year project working directly with young people to design and deliver interventions that increase demand for, and voluntary uptake of, modern contraception among adolescent girls aged 15 to 19 years. A360 designed and implemented four interventions across three countries – *Smart Start* in Ethiopia, *Kuwa Mjanja* in Tanzania, *Matasa Matan Arewa (MMA)* in northern Nigeria, and *9ja Girls* in southern Nigeria.

A360 adopted a user-centered approach throughout design and implementation, recognizing a need to evolve traditional approaches to adolescent and youth sexual and reproductive health (AYSRH) programming. Each A360 intervention leads with discussion of girls’ aspirations (inclusive of motherhood) and then positions contraception as a tool that can assist girls (and couples) to achieve their goals. In addition, A360’s approaches strengthen the health system to respond to the unique needs of adolescents and to provide adolescent girls with a full array of short- and long-acting contraceptive methods in a supportive environment.

The A360 investment was divided into three distinct project phases. These included an **inquiry phase** to understand the experiences, contexts, and underlying motivations that inform adolescent behavior; **insight synthesis and prototyping** by multi-disciplinary youth-adult teams; and **implementation**, beginning with an intentional period of intervention ‘optimization’ and grounded throughout in adaptation and ongoing quality improvement. A360 emphasized meaningful engagement of young people to co-design interventions that would be relevant within individual country contexts and continued to pursue continuous quality program improvement after design. Implementation in each country centered around an adolescent girl’s unique “user journey”, the term A360 uses to describe a girl’s experience with the project’s interventions. These user journeys and details of each A360 intervention are described in the project’s series of technical publications. By the end of its initial investment period in 2020, A360 had supported over 420,000 adolescent girls to adopt a modern contraceptive method of their choice, despite the last year being severely impacted by the coronavirus disease 2019 (COVID-19) pandemic. Over 40% of A360-supported adopters chose a long-acting reversible contraceptive (LARC) method.

The initial A360 program concluded in September 2020 and its follow-on phase will extend through 2025. A360’s follow on phase will be primarily implemented in northern Nigeria, Ethiopia, and Kenya. In this follow-on phase, A360 will continue to make adaptations to improve the effectiveness of its interventions and pursue integration of these interventions into government health systems. Alongside these priorities A360 will implement a comprehensive research and learning agenda that is crafted to address evidence gaps within the AYSRH community of practice.

This publication presents some of the key lessons from the initial A360 investment, implemented from 2016 to 2020. Lessons are oriented so that the reader can understand how A360 built on the evidence base (including its formative research), innovated in response to user insights during implementation, and learned throughout the process. These lessons critically informed the crafting of the technical strategy for the project’s follow-on phase. A360 maintains a commitment to transparency – about project successes and failures, both of which are emphasized in equal measure throughout this publication. In presenting these lessons, A360 aims to catalyze a conversation – among implementing partners, funders, public and private sector stakeholders, and young people – about how to address the persistent barriers which inhibit adolescents from pursuing their holistic aspirations. A360 welcomes dialogue with others who are keen to see meaningful progress is achieved.

## Key lessons learned

**1. Lead with girls’ aspirations to establish contraceptive relevance, and prioritize multi-sectoral programming**

*“I wanted to learn all these things [e.g., life skills, vocational skills, health knowledge] even before I heard of the program. When my mentor came and told me about MMA, I saw this as an opportunity to do so.”* –Former MMA participant; MMA Gender and User Journey Inquiry, Center for Girls Education 2020

***What does the evidence say?***

Adolescence is a vital period for defining aspirations and life goals related to education, livelihood, and family. Interventions that foster optimism about the future, positive self-esteem, and skills have all been shown to support healthy decision-making, including around contraceptive use ^10, 11^. Additionally, approaches that frame contraception as a tool to help achieve life aspirations as opposed to only focusing on mitigating risk, or method side effects, are similarly associated with increased modern contraceptive use among adolescents ^9, 12^.

During A360’s formative research, girls conveyed the expansive dreams they had for their lives that included, but extended beyond, motherhood. Even when they had sex occasionally, adolescents in A360’s target geographies often did not identify as sexually active for a variety of reasons, including stigma and perception of sex as a behavior not an identity. Traditional SRH approaches lead with sexual activity as a precursor to contraceptive use and often fail to acknowledge the central role that motherhood plays within girls’ aspirations. Since girls did not consider themselves sexually active and still maintained motherhood as a pivotal life aspiration, many did not perceive use of contraceptives as relevant to them.

***What did A360 do?***

**Figure 1.**
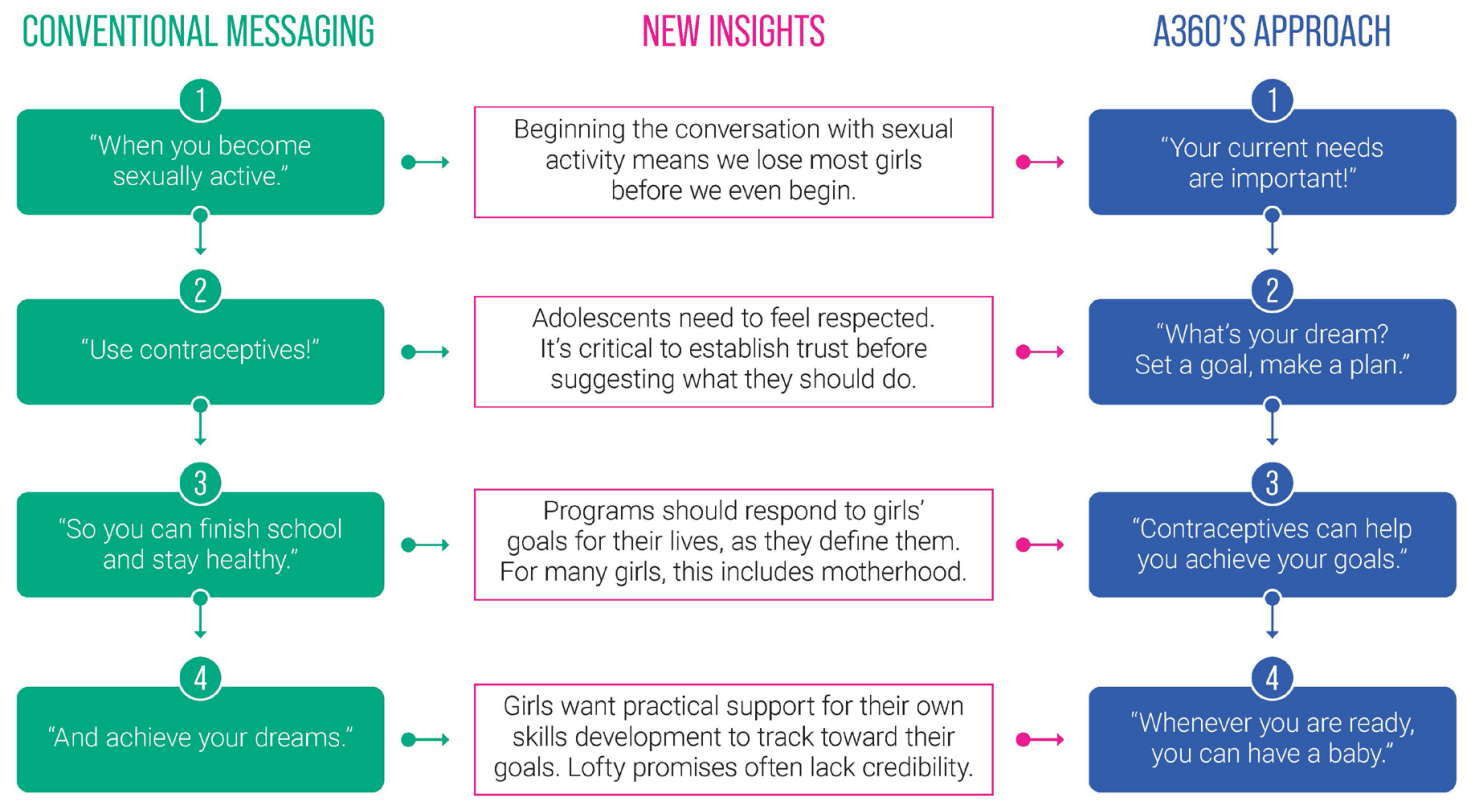
A360’s aspirational messaging compared to traditional sexual and reproductive health (SRH) approaches.

A360’s formative research helped the project to understand why conventional SRH messages did not resonate with adolescent girls in its target geographies. In contrast to traditional messaging, A360’s aspirational programming approach validates girls’ life goals and the full breadth of their aspirations, including educational and economic goals alongside motherhood. The project found this to be an effective way to make contraception feel applicable, while still emphasizing girls’ rights and agency ( Figure 1). The aspirational and skills building components embedded in each intervention model also capture girls’ interest and motivate them to engage with A360’s SRH programming.

A360’s aspirational programming components vary in intensity and scope across the project’s interventions given contextual and population-specific factors. They range from a financial planning exercise embedded within contraceptive counseling in Ethiopia, to vocational skills demonstrations in Tanzania, to life and vocational skills classes in Nigeria. Each component is intended to inspire girls to pursue broader aspirations in life, build tangible and applicable skills, develop their self-efficacy, and position contraception as a tool which could contribute to realization of their short- and long-term goals.

*“The mentor came to our compound to invite us, she told us that we will be learning about how to take care of our family, about nutrition, FP [family planning] but what got me interested was that she said at the end, we will learn a skill.”* –Girl, Nasarawa; Process Evaluation Nigeria, Itad 2019

***What did we learn?***

A360 reached over 650,000 adolescent girls with these low-intensity life and vocational skills-building components over the course of the project. These components were successful at supporting them to appreciate the relevance of contraception – three out of four adolescent girls who received contraceptive counseling through A360’s programming voluntarily adopted a modern method of contraception. These components also generated good will and buy-in within A360 implementation communities, garnering support among adolescent girls’ influencers for them to participate in SRH-related programming.

“ *What’s good about Kuwa Mjanja is the life skills element. …Considering also how the situation is with our leaders, when you come in straight away and say that you are just advocating about family planning directly, it may not be good. But, when you go in with the life skills, you can bring in the reproductive health education which will make it easier to get the support from our leaders from different levels*.” – National government stakeholder interviewed for A360 Tanzania process evaluation, Itad 2020

Despite the clear value of A360’s aspirational program components in leading to positive SRH outcomes, there were drawbacks to the approach which provided valuable lessons to A360 during implementation. Given the core objectives of the project were SRH-related, A360 acknowledges that the low dose of these components was sufficient to inspire, but not to truly empower. As a result, while some participants reported being able to apply their skills to earn income, this was an infrequent occurrence. Most referenced practical concerns, such as a lack of capital, insufficient time to learn and practice skills, and a mismatch between the skills provided and the available market, which limited their ability to apply their skills to earn income. The project also noted that the skills-building components tended to be overemphasized in mobilization messaging, leading to confusion and in rare cases backlash if community members did not sufficiently recognize that the core of A360’s programming was SRH-related.

Given A360’s SRH focus, the project did not introduce metrics to evaluate these aspirational components. The absence of metrics was a missed opportunity to capture the effect of these activities as standalone components and their contribution to the project’s SRH outcomes. At a minimum, incorporating metrics would have allowed the project to ensure that these components were meaningfully meeting the needs of participants.

***Where are we going next?***

A360 recognized that providing girls with more substantive support to achieve their financial and life goals could have multiple benefits and is aligned with global evidence on positive youth development (PYD). In its follow-on phase, A360 is working to improve the skills-building component within its SRH programming, within the constraints of program resources. This includes agreeing on the objectives and desired outcomes of these low-intensity skills components and monitoring these performance metrics, improving the relevance of the skills provided based on market assessments, and taking a rights-based approach to the content. In addition, A360 undertook a human centered design (HCD) process in 2021 to design further components to its models which expand support for girls’ economic strengthening and economic autonomy. These components will enter a pilot phase beginning in 2022.

**2. Program for an improved enabling environment to pursue gender-transformative impact**

***What does the evidence say?***

Biological sex and gender are important determinants of health ^13, 14^. Harmful gender norms and discrimination contribute to unequal access to resources and can impact decision making, mobility, access to health care, as well as health system response ^14, 15^. There is an inherently relational aspect in how adolescent girls make sexual and reproductive health decisions, involving interactions with others, particularly sexual or romantic partners but also family or community members and health system stakeholders ^16^. Gender and social norms play a significant role in these decision-making processes, governing and often limiting adolescent girls’ understanding of and ability to exercise power. Norm change is slow and can be met with significant resistance, particularly when those norms uphold systems of gender inequality ^16^.

Support from adolescent girls’ key influencers is central to fostering an enabling environment for their contraceptive use. Influencers such as male partners, parents, mothers-in-law, and religious or other community leaders can be facilitators or barriers to contraceptive uptake and continuation ^4, 17^. Approaches that focus solely on girls often fail to contribute sustainable progress towards gender equality ^18^. A360’s formative research validated the importance of engaging key influencers, and clarified a hierarchy of influence that different stakeholders had in girls’ lives – for example in Ethiopia, married girls identified male partners and mothers-in-law as key influencers but noted that when male partners were supportive, resistance from mothers-in-law was dramatically reduced. In A360’s formative research the project also found that the same messages that resonated with adolescent girls around pursuing a healthy and prosperous future also resonated with others in their communities.

***What did A360 do?***

**Figure 2.**
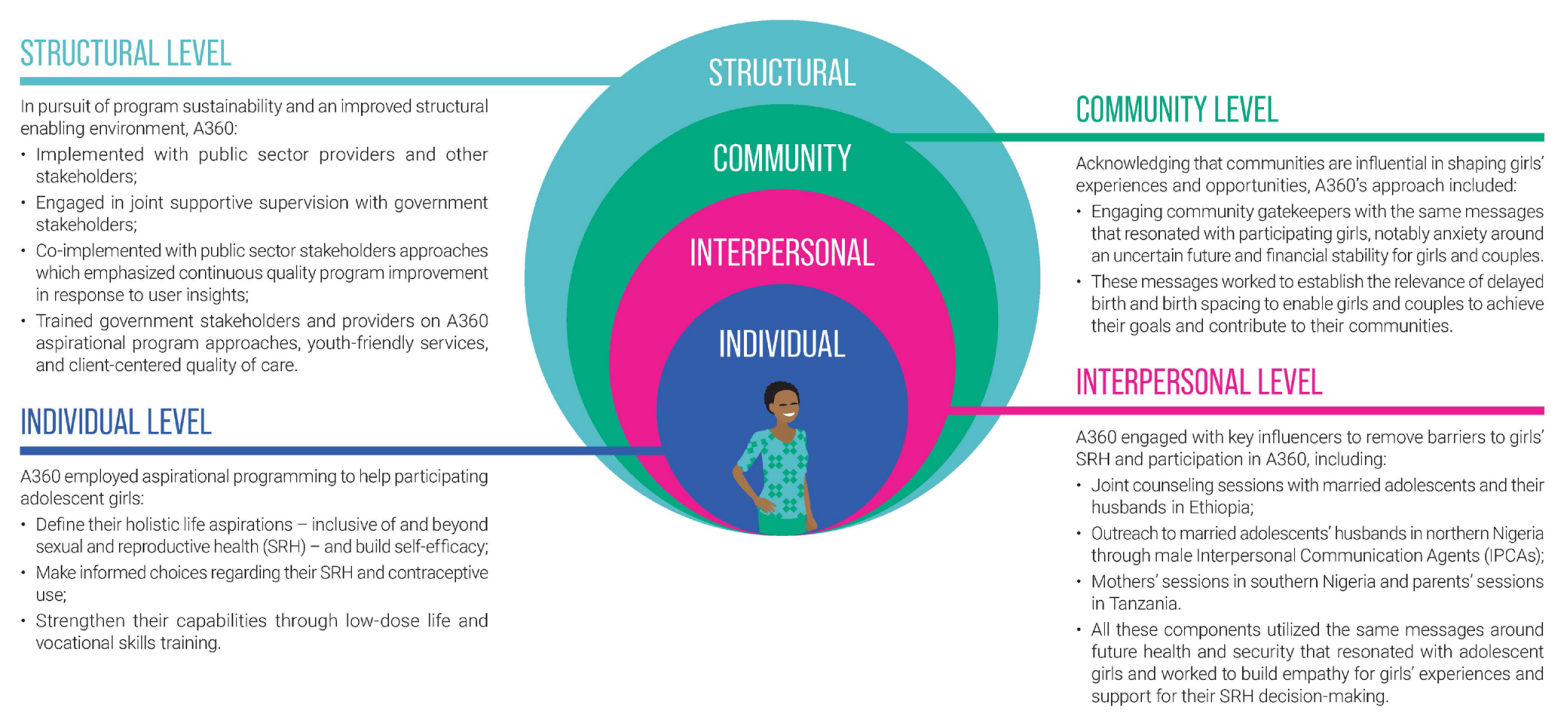
A360’s strategies for improving the enabling environment at each level of the socio-ecologic model.

A360 worked to design interventions which included components at each level of the socio-ecologic model that aimed to support girl’s agency and improve the enabling environment for their contraceptive use, within the constraints of the project’s mandate and resources ( Figure 2). The aspirational messaging developed through A360’s multi-disciplinary HCD process was leveraged to engage girls’ key influencers and community members. Directly asking girls to identify their key influencers during the formative research phase allowed A360 to maximize program resources, recognizing that targeting every influencer population would be too resource intensive. For example, in response to the insights from Ethiopian girls, A360 intensified engagement of male partners with the understanding that they could transform the attitudes of other key influencers. Building further on these insights, A360 engaged traditional and religious leaders as male partners themselves told the project that these leaders were influential in shifting their opinions.

“ *My husband was uninterested in birth spacing but after he met with the IPCA [interpersonal communication agent], he changed his mind. It would have been difficult for me to convince him, but he listens to other men*.” –Married girl, MMA participant
“ *It’s good to learn [about contraception] together. I’d want us both to know*!” –Husband, Oromia (Smart Start)

***What did we learn?***

A360’s learning reinforced that the enabling environment, particularly support from key influencers, is a significant determinant of whether adolescent girls adopt and continue to use contraception. This was particularly true of A360’s programming with married adolescent girls. Ethnographic research conducted by the Centre for Girls Education (CGE) in northern Nigeria on A360’s MMA program demonstrated that husbands were most often the primary decision-makers regarding whether girls attended events ^19^. If husbands were not provided with comprehensive information on the program’s content, they were often resistant to girls’ attendance as well as to their contraceptive use. In Ethiopia, Smart Start monitoring data showed that three out of five girls counseled without their husbands adopted a method of contraception, compared to four out of five when girls were counseled with their husbands ^20^. That said, husbands attended only about one third of these joint counseling sessions, indicating a need for more intentional approaches to engaging husbands for joint decision-making.

*“In our culture here, the men and husbands make family planning decisions for their wives.”* –Male partner, Process Evaluation Nigeria, Itad 2019
*“My husband wants me to have at least one child, before I will do it”* –Adolescent Girl, Nasarawa; Process Evaluation Nigeria, Itad 2019

Despite the importance of these enabling environment components, A360 was not consistent throughout its entire project lifecycle in how they were prioritized. In early implementation, during the project’s optimization period, an outsized focus on cost-effectiveness prompted A360 to pause or eliminate program components which didn’t contribute to immediate contraceptive uptake. A360’s mid-term evaluation (MTE) confirmed what the project had suspected – that these components, such as more meaningful engagement of girls’ key influencers, were critical for program impact. A360’s pivot in response to these findings, including re-introducing and strengthening these program components, is detailed in the project’s reflections on the MTE.

Though A360 devoted time and energy to understanding the social and gender norms which underpin girls’ behaviors and experiences, learnings from this project period emphasized that a more intentional and meaningful approach was needed to address the root causes of discrimination which limit adolescent girls from pursuing their aspirations. The lack of a cohesive global gender strategy, one that detailed how the project would support adolescent girls’ agency and work to shift inequitable power structures, was identified as a clear gap which needed to be addressed in its follow-on phase.

***Where are we going next?***

In its follow-on investment, A360 has elevated agency and the enabling environment as primary outcomes within the project’s results framework, intensifying efforts to meaningfully contribute to shifting harmful norms and power structures that limit girls’ opportunities and aspirations. This included pursuing HCD processes, beginning in late 2020, to strengthen meaningful male partner and community engagement approaches across its interventions in northern Nigeria and Ethiopia. In 2021, as a first step towards meaningfully mainstreaming gender considerations across the project, A360 undertook a collaborative process to develop a gender strategy which will guide a cohesive approach across all program geographies to support girls’ agency and empowerment and better measure gender equality outcomes. This strategy includes a roadmap towards strengthening the ‘gender muscles’ within A360’s structures, processes, and team which will be pursued over the course of the follow-on investment.

**3. Supporting contraceptive continuation is critical and must be tailored to the unique experiences of adolescent girls**

***What does the evidence say?***

Approximately 38% of women who adopt a reversible contraceptive method discontinue use within 12 months, and over half (55%) discontinue within two years ^21^. Adolescents’ patterns of contraceptive use differ from all women of reproductive age and they may be likely to start and stop use more frequently due to intermittent sexual activity ^17^. Evidence also suggests that adolescents may discontinue at rates 25% higher than those for older women ^22^. Method-related side effects are a leading cause of discontinuation, but other drivers particularly for adolescents include changing reproductive needs (i.e. period of abstinence), changing reproductive intentions (i.e. a desire to become pregnant), and hesitation to seek contraceptives due to a negative experience with a healthcare provider, inconvenience, access, cost, and stigma ^21, 23^. Despite this knowledge, there is a distinct lack of evidence around what works to support adolescent girls and young women to use contraception in line with their unique patterns of sexual activity and need, and to reduce discontinuation while they are still in need of contraception, particularly which approaches are scalable and able to be sustained within existing health structures.

***What did A360 do?***

A360’s early implementation phase (2018) was characterized by an overemphasis on contraceptive uptake as the primary metric of program success and a lack of focus on support for adolescent girls’ continued contraceptive use while still in need. In this phase, A360 focused on program adaptations that improved contraceptive uptake and saw tremendous results, increasing contraceptive adopter numbers four-fold from the first quarter to the last quarter during the first year of implementation. However, A360’s analysis of performance data, external evaluation results at midline, and tracing of small cohorts of girls in Ethiopia and Nigeria all reinforced the need to consider factors beyond contraceptive uptake to promote broader impact. As a result, the project tested a series of small adaptations to support adolescent girls who adopted a contraceptive method through A360 interventions to continue using contraception while still in need, including to switch methods. These adaptations included supporting public providers to apply client-centered counseling methodologies using PSI’s Counseling for Choice (C4C) ^ii^ approach, instituting client follow-up systems (both through physical tracing of clients and through an outbound call center), exploring community-based distribution of self-injectable contraception (DMPA-SC), and providing access to on-demand information and referrals through an unstructured supplementary service data (USSD) portal. ^iii^

***What did we learn?***

A360 learned a great deal regarding the factors that shape girls’ decisions around continued method use, reinforcing some of the same challenges that the broader AYSRH sector is wrestling with. There is some indication that A360 adopters are continuing method use at rates higher than those demonstrated in the evidence base. In Ethiopia, for example, data from a sample of sites where A360 transitioned implementation to government indicates 12-month continuation rates of around 60%. Additionally, there is compelling evidence of high-quality youth-friendly service delivery and positive relationships between providers and youth in A360 sites that are effectively supporting continuation ^20^. For example, in northern Nigeria nearly all client exit interview (CEI) respondents said they were informed about alternative methods that they could use, 90% said they were informed about side effects, and 87% about what to do if they experienced side effects. Four in five girls said they understood that they could switch methods if they needed or wanted to do so ^24^.

*“Since I was counselled, I know if anything should happen, I have a place to come…I know that if I don’t want to get pregnant, I should come here and I will be protected.”* –Girl, Ogun; Process Evaluation Nigeria, Itad 2019

Still, A360’s learning echoes the global evidence – contraceptive side effects remain a primary driver of discontinuation. Girls interviewed for the project’s process evaluation in Ethiopia and northern Nigeria highlighted that side effects including fatigue, headaches, and heavy bleeding were their primary reasons for discontinuing method use even while still in need of contraception.

While intensifying support for management of side effects undoubtedly remains important, A360’s learning also reinforced the necessity of strategies to promote continuation when in need that extend beyond the provider-client relationship. These include strategies that address community and familial norms around contraceptive use. Adolescent girls’ ability to use contraception in line with their fertility preferences relies on a supportive enabling environment. In-depth interviews with A360 adopters who discontinued contraceptive use revealed a heightened sense of stigma around modern contraception and a lack of support from husbands and other influencers such as mothers and mothers-in-law. For girls who discontinued and their influencers, the presence of side effects often served to validate pre-conceived fear and misconceptions, for example, reinforcing perceptions that contraceptive use can threaten future fertility. In some cases, side effects were used by key influencers who were not supportive to pressure girls to discontinue use while they still had a need for contraception ^20^.

“ *[A girl who uses contraceptives] might age and not get the child when she wants it.*” –Mother of adolescent girl, Tigray; Process Evaluation Ethiopia, Itad 2019

A360’s learning reinforces that adolescent sexual activity is often unplanned and infrequent ^22, 25^ (combined with the prevalence of non-consensual sex). Strategies to support continuation must respond to these unique patterns of contraceptive need and use, effectively assisting adolescents to start, pause, and resume contraceptive use according to circumstance and need.

***Where are we going next?***

**Figure 3.**
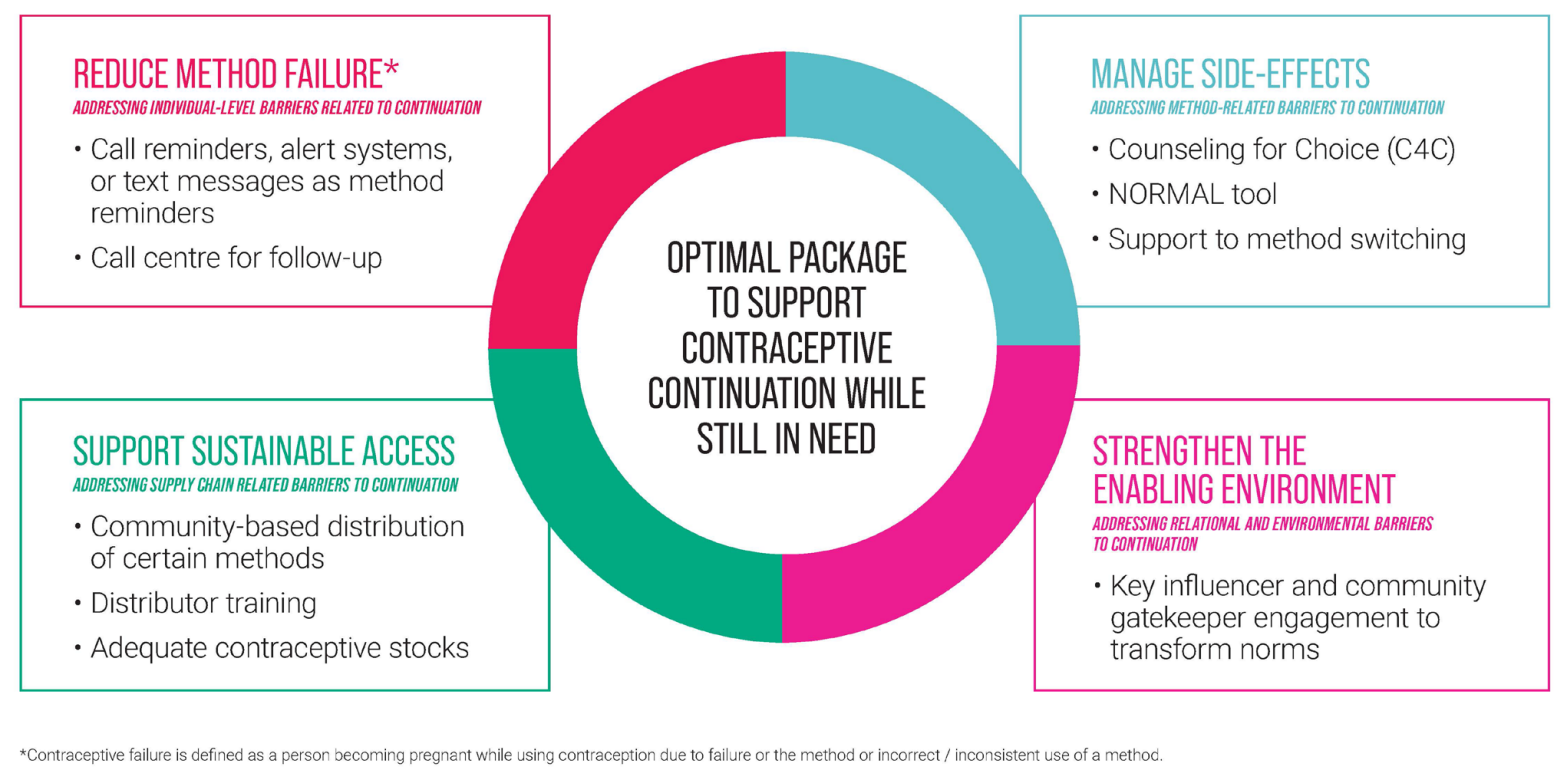
A360 optimal package approach to support contraceptive continuation when in need.

 A360 combined learning from its first investment along with a comprehensive review of the literature to shape its continuation strategy under its follow-on investment. The lack of global evidence on patterns of contraceptive use among adolescents presents an opportunity to explore what is driving the decisions that adolescent girls and couples make to continue using contraception or not, and to identify what initiatives, or combination thereof, are most effective in supporting continuation in these contexts. A360 is planning continuation cohort studies across two of its geographies – Nigeria and Kenya – to generate evidence on adolescents’ unique patterns of use and drivers of discontinuation. A360 developed an optimal package consisting of four strategies for supporting continuation, built on evidence-based best practices ( Figure 3). A360 will test out this optimal package to see which components are most effective at supporting girls to continue to use contraception while in need and will scale up those approaches which are shown to have the greatest impact.

**4. Maintain a commitment to learning and continue to iterate beyond design**

***What does the evidence say?***

Global evidence is clear – interventions benefit from ongoing continuous quality improvement during implementation to refine and strengthen them in response to changing contexts and emerging opportunities. Evidence collected in a narrow, relatively optimal set of circumstances, such as design, may not apply in the same way in every implementation context. In other words, design creates an incubator for positive outcomes, but interventions can never be “optimized” prior to implementation in an actual, real-world setting ^26^. Approaches which involve refining and adjusting during implementation are more effective than ‘quality assurance’ approaches which emphasize fidelity ^iv^ to the initial intervention design.

***What did A360 do?***

As A360 began implementation in 2018, it incorporated adaptive implementation to retain its user-centered focus, while continuing to refine its interventions post-design. A360 embraced the need for iterative, learning-based implementation to identify areas where the project could be strengthened to respond to the needs of adolescent girls and local health system actors, while maintaining fidelity to core foundational elements of its interventions. The project designed monitoring and field research systems that allowed for rapid review of qualitative and quantitative data to inform continual adaptation with a multi-disciplinary group of stakeholders. As the project moved from design to implementation, HCD transitioned from the primary discipline to a supporting one, enhanced by youth-led participatory action research (YPAR) approaches ^27^. Adaptive implementation supplied a framework for understanding performance and supported country teams to introduce adaptations to improve intervention effectiveness. Young people played a significant role during the adaptive implementation phase, with youth program staff often identifying and rapidly testing small prototypes to improve performance and responsiveness.

To assist decision-making, A360 developed a set of adaptation guidelines that articulated which intervention components *should* be preserved with fidelity and those which teams felt could be carefully adapted without compromising their effectiveness. An annual "adaptation audit" was carried out with each program team from 2018 to 2020 to reflect on the adaption process across intervention components—assessing when and how fidelity to the original design was maintained, documenting where adaptations were needed to strengthen the intervention, and identifying any points where the intervention approach may have drifted too far from its intended principles.

***What did we learn?***

A360 found that adaptive implementation allowed the project to continue using a ‘design mindset’ as it moved from its formative research phases into implementation. The project came to understand that though HCD and adaptive implementation are different in their methodological approaches, they share a few key traits. Both are guided by user’s voices, perspectives, and experiences; can be conducted in partnership with users, government, and other partners; and are informed by the global evidence base and a variety of disciplinary lenses. Both cultivate a culture of curiosity and inquiry to inform improvement through iteration, and orient teams to the use of data and field-based research. The skills and mindsets fostered through HCD (flexibility, curiosity, and the ability to test and to iterate) helped A360 project teams adjust to and apply an adaptive implementation approach. For programs that do not have the resources to apply full HCD processes from program start to finish (considered ‘HCD end-to-end projects’) ^28^, adaptive implementation can be critical in maintaining curiosity and flexibility throughout the course of implementation.

Adaptive implementation yielded considerable value for the program. It created new insights, deeper and more rigorous understanding of these insights, as well as shaped refinements to ensure the interventions ‘worked’ in the context of real-world implementation. It enabled A360 to monitor fidelity to its unique user journey while leaving room for intervention improvement. By engaging local government counterparts in the process, A360 was able to build their capacity to understand how to deliver adolescent-responsive contraceptive services.

Despite adaptive implementation approaches yielding considerable benefits, A360 identified distinct opportunities for improvement. The framework that the project used to guide adaptative implementation processes was not always intuitive to project teams. As a result, teams did not always apply a systematic approach to adaptive implementation, often the result of a lack of tools and understanding around which components were truly ‘core’ to program effectiveness and needed to be faithfully preserved. Space to adapt and to evolve needs to be accompanied by a clear process and parameters to determine what gets adapted and how, along with indicators, to make efficient use of finite time and resources.

The curious and iterative project culture which was nurtured through HCD and adaptive implementation also influenced A360’s organizational structure and processes. The foundation built from HCD and adaptive implementation allowed A360 to embrace adaptive management and governance – maintaining flexibility to shift budget, staffing structures, and ways of working to accommodate each new project phase. The mindset which was established from HCD and adaptive implementation emphasized continuous learning and program improvement. This mindset extended beyond A360’s interventions, prompting the project to institute intentional pause and reflect moments to consider whether all aspects of the project management and governance continued to be ‘fit for purpose.’

***Where are we going next?***

 This current phase of A360 offers an opportunity to improve upon the adaptive implementation process from the initial project period – simplifying, refining, and strengthening its fit for purpose to increase its use within the project. A360 revamped its adaptive implementation framework and tools which were rolled out in late 2021. The framework will promote a more simplified and systematic approach to adaptive implementation and will support project teams to routinely document adaptations rather than relying on annual retrospective adaptation audits. This framework is being intentionally crafted to be fit for purpose, not just for A360, but also for application by government counterparts and other players in the AYSRH sector. A360 plans to publish a final version of this framework externally in 2022.

**5. Design with the end in mind: pursue sustainable scale from the outset**

***What does the evidence say?***

Realization of global commitments, such as the sustainable development goals (SDGs), highlights that universal access to SRH services, including contraception, will only be achievable if “promising and proven service improvements” are introduced more widely and small-scale projects are expanded ^29^. However, a “present bias” ^30^ persists, especially in many donor-funded programs with relatively short time horizons. Despite efforts by global governing bodies such as the World Health Organization (WHO), USAID, ExpandNet, Family Planning High Impact Practices (HIP), and Implementing Best Practices (IBP) to provide guidance on evidence-based practices that promote sustainability, there are limited precedents of multi-component SRH programs successfully scaling ^4^, with sustainability often remaining conceptual rather than actualized.

***What did A360 do?***

Throughout its initial investment, A360 implemented its intervention models through existing public health structures and in partnership with government actors. The project intentionally focused on public structures both in response to user preference as well as to provide the greatest chance for sustainable scale. A360’s adaptive implementation and supportive supervision processes involved multi-disciplinary teams made up of various public sector stakeholders. Services were provided primarily by public sector providers in public facilities. Despite this intense engagement with government, A360’s intervention models were not explicitly designed for full government institutionalization.

**Figure 4.**
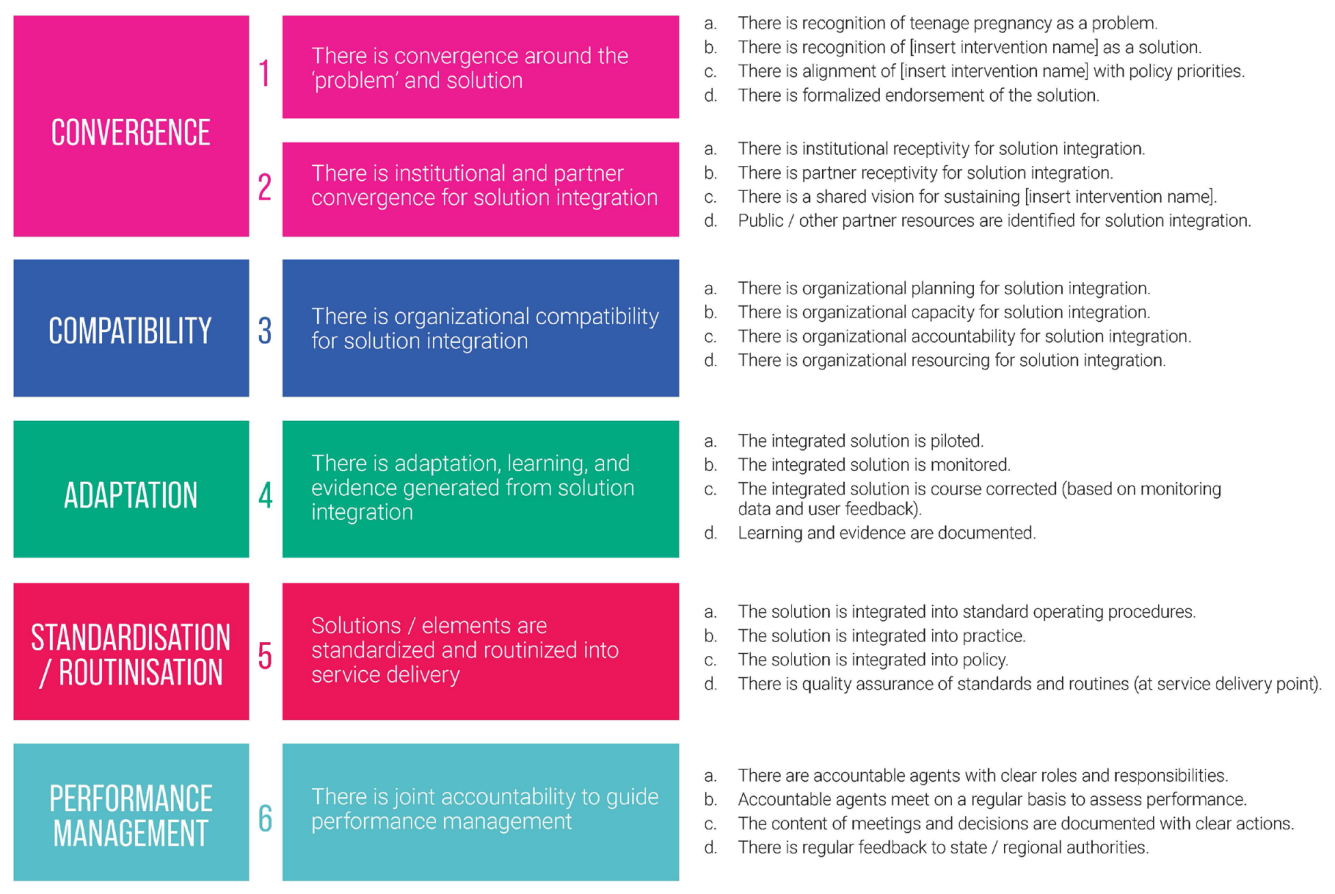
Government integration milestone framework.

After completing a year and a half of implementation, strengthening its intervention models through adaptive implementation, and generating preliminary evidence on its intervention effectiveness, A360 intensified its focus on sustainability. This focus acknowledged that the primary avenue for sustainability for the project’s intervention models would be through integration within government systems. In the latter half of 2019, A360 began assessing the readiness of its intervention models for government integration, partnering with ExpandNet and other technical experts. This process included development of an integration milestone framework ( Figure 4) which could be used to assess progress towards integration. The framework identifies five critical domains along with associated milestones that are important for the project to achieve in pursuit of sustainability. This framework supported project teams to identify manageable steps and criteria towards achievement of sustainability goals, to agree on milestones with government to track incremental progress towards sustainability, and to inform accountability mechanisms for A360 and government stakeholders.

***What did we learn?***

A360’s experience reinforces the importance of designing with an eye towards sustainable scale-up. An intensive focus on design research to understand girls’ desires and aspirations was imperative for the project but needed to be balanced with a focus on the health system actors who would ultimately be the frontline implementers. These actors warrant attention, not only because they deserve empathy, but because resonance of the interventions with their own intrinsic motivations is vital for implementation success.

At the same time, A360 grappled with a critical question: *How do we engage in a conversation about sustainability before an intervention is proven effective*? Evidence from the project’s external evaluation of the first phase suggested that focusing on adoption and replication of program solutions *before* they were proven (and had undergone adaptive implementation) may have been premature. Designing for the individual client must take priority. However, projects looking to design interventions that are both user-centered and sustainable must wrestle with a decision around *when* interventions are considered adequately proven to merit pursuit of sustainable scale.

***Where are we going next?***

A360 is committed to evaluating the scalability and sustainability of its interventions. Integration into government systems is key to responsive adolescent programming that can be sustained after the program concludes. As part of its strategy for the follow-on investment phase, A360 mapped out a phased plan for government integration for its interventions in Ethiopia and Nigeria. This plan began with a period of adapting A360’s existing interventions for fit within government systems. Part of the exercise with ExpandNet to assess replicability involved mapping the resource requirements of A360’s intervention components against existing resources within government health systems. Service delivery components, such as supportive supervision, and provider training, were deemed to be highly aligned with existing health structures and thus could be more readily integrated. By contrast, some intervention components were more nuanced and difficult to integrate, like the aspirational and vocational skills components.

The process for integration differs across Ethiopia and Nigeria depending on the level of health system decentralization. Ethiopia has a nationally centralized system. In 2019, the Federal Ministry of Health committed to rolling out Smart Start nationwide commencing at the national level and then cascading to lower levels in the health system. Nigeria is decentralized to the state level and so A360 is integrating MMA into Kaduna, Nasarawa, Jigawa, and Kano state governments and scaling to all viable local government areas (LGAs) in those states. A360 will apply implementation research to generate evidence on the barriers and facilitators to integration of A360’s interventions into government systems in conjunction with this phased process of integration.

## Conclusion

A360’s initial project phase generated a tremendous amount of learning. A360’s **aspirational program components** were critical to the project’s success at engaging adolescent girls and providing them with relevant, effective services. Yet, there is clear value in expanding these components to meaningfully support girls to pursue their economic and life goals, acknowledging that these components will reach a smaller scale than A360’s broader SRH program given the significant resource needs of economic strengthening programming for adolescents. Programming for an **improved enabling environment**, and specifically involvement of girls’ key influencers and communities, is critical to transformation of inequitable norms that limit girls’ SRH and agency. A360 engaged in strategies that targeted all levels of the enabling environment but recognized the need for a more consistent and rigorous approach that could be truly transformative. **Contraceptive continuation** among adolescents remains a challenge for the global AYSRH community of practice. A360’s learning reinforces a need to find strategies or a package of services that respond to adolescent girls’ unique patterns of sexual activity, which are often more intermittent – and measurement approaches which are relevant and effective at understanding whether girls are able to continue to use contraception in line with their fertility intentions and aspirations. Continuous quality program improvement through **adaptive implementation** critically supported A360 to retain its user centered focus during implementation and created a culture of curiosity and iteration that also influenced the project’s governance and global structures. Lastly, A360’s learning highlights valuable questions related to **program sustainability**. Designing for end users must be balanced with design for the systems and structures through which an intervention will be implemented. However, at what point can programs adequately prove their interventions are effective enough to justify pursuing sustainable scale-up, particularly through government integration? These lessons (and questions) have guided A360 in the development of its technical strategy under its second phase. Yet, the need for learning and iteration has not stopped. A360 continues to grow in its understanding of what it takes to support sustained, transformative, holistic change for adolescent girls and commits to openness and transparency regarding successes and failures during its next project phase.

## Data availability

No data are associated with this article.

## Footnotes

^i^ A360’s design phase consortium included IDEO.org, University of California at Berkeley Center on the Developing Adolescent, and Triggerise. The project’s implementation partner in Nigeria is the Society for Family Health Nigeria. A360’s external evaluation partners included Itad, the London School of Hygiene and Tropical Medicine (LSHTM), and Avenir Health.

^ii^ Counseling for Choice (C4C) is a PSI initiative that contributes to a positive narrative around contraceptive choice. C4C pulls from existing counseling best practices and is intended to maximize client satisfaction and reduce method discontinuation among girls and women in need of contraception.

^iii^ Through a free USSD portal, accessible on any mobile phone, adolescents could access customized SRH information via text that responded to their specific needs. This included responses to commonly asked questions about contraceptive use, contraceptive side effects, and menstruation.

^iv^ Fidelity references strict preservation of an intervention according to its original design, without exception.
