# Supplementary material for: Implementing adaptive youth-centered adolescent sexual reproductive health programming: learning from the Adolescents 360 project in Tanzania, Ethiopia, and Nigeria (2016-2020)
Source: Gates Open Res. 2022 Mar 25;6:14. [Version 1] doi: 10.12688/gatesopenres.13589.1 (PMC9110738; doi:10.12688/gatesopenres.13589.1)
Supplement: Peer review history

# Peer review: Reviewer response for version 1

**DOI:** 10.21956/gatesopenres.14859.r31922

**Published:** 2022-05-16

**Authors:** Adriane Martin Hilber

**Affiliations:** 1 Swiss Tropical and Public Health Institute (Swiss TPH), Basel, Switzerland

The article reports on the first phase learning, and first 2 years of a transition phase which began in 2020 of the Adolescent 360 program in Nigeria, Ethiopia and Tanzania. It reviews the A360 investment across 3 distinct project phases (inquiry phase, insight and prototyping phase and implementation phase) to draw out 5 key lessons learned. As much of the program has been described in other publications, the article summaries findings and lessons learned with reference to the larger descriptive analysis of the phases or specific interventions strategies. While this is understandable given the breadth of information they wanted to cover by the article, the reader is sometimes left wanting to understand and appreciate from where the learning came in greater detail and context. This was the case, however, in a minority of lessons and observations (see comments below). Generally, the observations and lessons learned are well grounded in the global evidence, as well as the results of the country level activities from design to implementation and evaluation. As a result, the article offers a wealth of insights that can inform the field, as well as the further implementation of the project. Some of the ambitions however of A360 may not be achieved as they are numerous, and may very well exceed the time and funding available to see sustainable scale up as envisioned - but this of course, is the case with most projects. A360 achievements and learning to date are impressive and well worth following as they proceed through the next phase of implementation and evaluation.

 Specific Points:

 Background: The section adequately explains the program and its phases. It discusses adolescent meaningful participation and co-design to better understand the user journey but does not elaborate how this was done. In the technical brief's meaningful participation is again highlighted but not explained. *It would be interesting for the project to reflect on how they have engaged adolescents and the lessons learned from that experience which they seem to have done through the phases of the project.*

 The **background (p3-4)** and descriptions of the lessons learned say little about the legal and policy context which underpinned implementation undoubtedly. *It would be interesting to know how laws and policies (related to contraception for example) affected the program in each country either in the background section briefly or within the lessons elaborated.*

**Key Lessons learned:**

 The organization of the report is easy to follow and helpful.

- *Sometimes, the text strays from the purpose of the subsection which could be streamlined.* For example in Lesson 1 on "what does the evidence say?", the second paragraph goes into findings from the formative research for the program. This information should lead in the next section (What A360 did) or be well referenced in the evidence section. Currently there are no reference for the observations from the formative research. (p4).
- The graphic to share the A360 approach is clear and helps the reader to better understand the A360 approach. (p4).
- "What did we learn?" (p5) - the section explains the drawbacks which are interesting, particularly related to the low dose of skills building which "inspires" but is not "empowering". The mismatch lead to disappointment and in some cases backlash, it states. In the "Where are we going next" section however, there is no mention on how such backlash will be addressed (or was addressed). It simply states more work is needed on skills building but does not say what is being proposed. It would be interesting to know how such limitations are proposed to be practically managed given the constraints of projects. This would be an important learning to detail. Currently the explanation balance setting goal posts lower (agree on low intensity skills), measure better, and then, they will design better economic models which seems to have already started. More information on what is proposed would strengthen the section.
- Lesson 2: Here again on the evidence, formative research is cited. Maybe add a subsection to differentiate between global evidence and project evidence in these sections (p5).
- This section is more detailed on the learning and explanation with another nice graphic detailing the findings and reference to the brief on reflection on the MTR. *It then goes on to say more time was devoted to gender and social norms but does not say what the specific problem was (what root cause of discrimination?) that a new strategy was to respond to. It gets a bit cryptic. More detail here would be welcome. (p7). *
- Lesson 3:The evidence section highlights that there is a lack of evidence around what works to support AGYW to use and stay on contraceptives, but the "what you will do" section does not adequately say how you will robustly address this knowledge gaps based on your analysis of existing monitoring and evaluation (MTR) data. The interventions to address the issues (contraceptive uptake) appear to be expensive and not sustainable. *It would be helpful to understand why those interventions were selected when they do not fully align with the underlying aim of the project to make the interventions sustainable by the government eventually. More explanatory detail is needed here. (p7).*
- In the "what we learned section" good detail is provided from your M&E data and publications which is helpful. The discussion on side effects and how to manage perceptions of them is particularly interesting (p8) as is the plan for continuation of the cohort studies to understand patterns of use. *The optimal package deserves an article in its own right, if not already done.*
- Lesson 4: The lesson as written is not as strong as it could be. *The issue is iteration and adaptation through implementation science. Adaptive implementation is the learning as well described. I suggest changing the title of the learning to better reflect that.* I particularly like the "pause and reflect" moments which is rare in project cycles but so needed. (p9).
- I appreciate the learning extended to project organization, staffing and processes. *It would be helpful to give some examples as those issues are rarely detailed as learnings.*
- Lesson 5: I appreciate the focus on scalability and the framework for government integration, however, how this should be done may need further reflection. The learning subsection asks important questions which seem to not be resolved yet. *The Mapping in Nigeria with ExpandNet seems promising but given the number of efforts that have done similar actions, it may be worth reviewing past efforts in Nigeria (or whichever context) specifically to understand and mitigate political barriers to expansion and scale up. A political economy analysis and or power analysis of the players and their interests could possibly help to situate the integration more effectively. I miss the political and regulatory lens to the learning which often can make or break uptake at country level. (p11).*
- Reference 30 on "present bias" could be updated. (p10).

Does the article adequately reference differing views and opinions?

Yes

Are all factual statements correct, and are statements and arguments made adequately supported by citations?

Yes

Is the Open Letter written in accessible language?

Yes

Where applicable, are recommendations and next steps explained clearly for others to follow?

Yes

Is the rationale for the Open Letter provided in sufficient detail?

Yes

Reviewer Expertise:

SRHR  and health systems implementation projects in development countries, particularly Africa; Implementation Science, Accountability in RMNCAH programs evaluation

I confirm that I have read this submission and believe that I have an appropriate level of expertise to confirm that it is of an acceptable scientific standard.

# Peer review: Reviewer response for version 1

**DOI:** 10.21956/gatesopenres.14859.r31921

**Published:** 2022-05-05

**Authors:** Rebecka Lundgren

**Affiliations:** 1 Center on Gender Equity and Health (GEH), University of California San Diego School of Medicine, La Jolla, CA, USA

This open letter presents the experiences and key lessons from a 4.5-year project working with young people to increase demand for, and voluntary uptake of, modern contraception among adolescent girls aged 15 to 19 years. This analysis stems from reflections during its transition into a follow-on program. A hallmark of this project was the use of human centered design (HCD) to create four adolescent sexual and reproductive health (ASRH) interventions across three countries, combined with the application of adaptive management approaches.

 The letter is organized around five lessons learned: 1) the value of aspirational program components and the need to meaningfully support girls to pursue their life goals holistically; 2) the necessity of taking a consistent and rigorous approach to improving the enabling environment for contraceptive use to promote transformative change; 3) the need to find program and measurement approaches that respond to girls’ unique patterns of sexual activity, and support contraceptive continuation; 4) the usefulness of continuous program improvement during implementation to maintain a user-centered focus and create a culture of curiosity and innovation; and 5) the tension between designing for users and beginning with program sustainability in mind from the outset.

 This is a valuable narrative of A360’s experiences, with lessons learned that have the potential to advance the field, if applied. The organization of the letter by key lessons, each with a section on evidence, A360 actions, learning and next steps, is excellent. The key lessons resonate with my knowledge of the experience of other projects, and the challenges, and approaches for addressing them represent critical learning for our field. The language is accessible and the figures supporting each key lesson well done and helpful.

 The challenge of this type of document, stemming in part from its required brevity, is providing adequate evidence to substantiate the assertions. It is not always clear what type of evidence backs up the authors’ assertations – is it research based – if so, what type? Is it program/experiential learning? If so, how was it collected? I recognize it is very difficult to add specifics for each lesson, but wherever some brief information could be added, it would be useful. The quotes used to support the learnings are informative, but don’t really provide a basis on which to assess the lessons learned. In some places, this was done very well.  See, for example, “what did we learn” for Lesson 3 (pg. 7). That type of evidence is surely not available for all the lessons, but any additional clarity where possible would strengthen the authors’ arguments.

 The authors did an excellent job discussing areas that did not work as well as desired. I commend the author’s commitment to transparency – their openness accelerates our ability to learn from their work. For example, see the discussion on p. 5 on the pros and cons of the skills building component, and the lack of metric. In this case, the evidence base is clear. I also appreciated the discussion (p. 6) of the “outsized focus on cost-effectiveness” and inconsistent focus on the enabling environment. These are widespread challenges.

 There are some general statements about what the field knows that could perhaps be better supported by additional citations. I suggest that the authors review the paper and add citations where possible. For example, the first paragraph on p. 5 under Lesson 2 seems vague and the reference not adequate. I’m calling this one out because it is a cornerstone of this work. As another example, the statement on p. 8, “global evidence shows contraceptive evidence a primary driver of discontinuation”, while broadly accepted could still use a citation.

 The recommendations are clear and important, although some are a tad obvious, and what is most needed is some guidance on how to achieve them. I suggest the authors look at each recommendation to see if there are any areas where the “how” could be strengthened with an additional sentence or two. See, for example, the discussion under “where we are going next” for Lesson 2 (p. 7) referencing applying HCD, a gender strategy and engaging men. All good recommendations but would be more meaningful with something more specific. Also on p. 9, Lesson 4 – an example or more detail on what “adaptive implementation” in the face of limited resources might look like would be helpful. Similarly, in the paragraph on p. 9 starting with “the curious and iterative project culture”, the recommendations are too vague to put into practice. As a final example, on p. 10 (Lesson 5), the statement “intervention models were not explicitly designed for full government institutionalization” is very important and a common challenge. Could you add an example of what was problematic?

 To wrap up my comments on this excellent work, I have thoughts on the background and conclusion sections. In the Background section, there was a bit of a disconnect for me between the first and second paragraphs. As many of us do, the authors walk a difficult tight rope between a focus on adolescent intentions and desires and a health and demographic justification for increasing CPR. The statement focusing on the gap between age at first sex and first contraceptive use especially struck me. The next paragraph goes into health explanations, but the first paragraph gives a very outsider point framing of the problem and seems to overlook the fact that young people may desire children or have other reasons for this gap. Given that the letter strongly addresses social and cultural context, I wonder if you might edit that paragraph, so it provides a better foundation for your excellent work. Finally, the Conclusion statement states that the aspirational program components were critical. This assertion didn’t resonate for me as I didn’t feel the evidence presented supported such a strong statement.

Does the article adequately reference differing views and opinions?

Yes

Are all factual statements correct, and are statements and arguments made adequately supported by citations?

Partly

Is the Open Letter written in accessible language?

Yes

Where applicable, are recommendations and next steps explained clearly for others to follow?

Yes

Is the rationale for the Open Letter provided in sufficient detail?

Yes

Reviewer Expertise:

Implementation research in the area of adolescent, sexual and reproductive health, with a focus on social norms, scale and sustainability.

I confirm that I have read this submission and believe that I have an appropriate level of expertise to confirm that it is of an acceptable scientific standard.
